# Fragile heroes. The psychological impact of the COVID-19 pandemic on health-care workers in Italy

**DOI:** 10.1371/journal.pone.0242538

**Published:** 2020-11-18

**Authors:** Chiara Conti, Lilybeth Fontanesi, Roberta Lanzara, Ilenia Rosa, Piero Porcelli

**Affiliations:** 1 Department of Psychological, Health, and Territorial Sciences, University “G. d'Annunzio” of Chieti‐Pescara, Chieti, Italy; 2 Department of Dynamic and Clinical Psychology, “Sapienza” University of Rome, Rome, Italy; Iwate Medical University, JAPAN

## Abstract

This survey-based study aimed to explore the mental health status and psychological care needs of 933 health-care workers in Italy during the COVID-19 outbreak. Sociodemographic data, exposure to COVID-19, perception of psychological care needs, depression, anxiety, somatization, and post-traumatic symptoms were concurrently assessed. The majority of the sample (71%) suffered from somatization and 55% of distress. Female care workers experienced higher levels of anxiety (d = 0.50) and somatization symptoms (d = 0.82) and stated they needed psychological care more than men (p < .001). Younger participants (aged <40 years-old) reported higher levels of somatization, depression, anxiety, and post-traumatic symptoms (effects size range from d = 0.22 to d = 0.31). Working in a high infected area (red-zones) and directly with COVID-19 patients (front-line) affected the psychological health of participants to a smaller degree. Health-care workers who lost one of their patients reported higher levels of depression (d = 0.22), anxiety (d = 0.19), post-traumatic symptoms (d = 0.30), and psychological care needs than those who did not have the same experience (p < .01). Health-care workers who perceived the need for psychological support scored above the clinical alarming level (cut-off scores) in all the psychological scales, ranging from 76% to 88%. Psychological distress (p < .01), anxiety (p < .05), depression (p < .05), and being women (p < .01) contribute to explain the need for psychological care and accounted for 32% of the variance in this sample. These findings point out the importance to consider the psychological impact of COVID-19 on Italian health-care workers and strongly suggest establishing psychological support services for providing adequate professional care.

## 1. Introduction

Since December 2019 in the Hubei province of China, the novel coronavirus disease (COVID-19) has spread rapidly worldwide [1, 2]. COVID-19 is a complex respiratory disease, characterized by human-to-human transmission, asymptomatic carrier transmission, high transmission efficiency, and involvement of multiple organs [1, 3]. The disease caused by the virus was considered a public health emergency by the World Health Organization and was declared a pandemic by March 2020 [4].

The first full-blown outbreak in Europe happened on 22nd February in Italy [5, 6] and consequently the disease spread worldwide with almost 37 million cases as of 13th October 2020 [6]. At the time of this survey, the total number of cases in Italy was exceeding 233,000 people, and the number of people killed by COVID-19 (33,530) outweighed the deaths recorded in China (3,259) [7]. To date, the total number of cases in Italy continued to increase, exceeding 359,000 cases, with 36,205 people killed by COVID-19 [6].

About 33,040 health-care workers (HCWs) have been infected and 113 have died during the epidemic in Italy [8], due to close contact with infected patients. Previous studies conducted in China reported that HCWs with a higher burden of workload and treating patients with life-threatening medical conditions were experiencing more severe psychological pressure, even psychopathological conditions [9]. Due to increasing patient volumes, medical professionals were recruited to support the front-line [10] even if not specialized in infectious diseases and may experience even greater pressure when facing infected patients [11]. To date, no definite treatment is available for this infection and no vaccines have been developed yet [12]. As already demonstrated during the severe acute respiratory syndrome (SARS), all these factors contribute to the increased immediate and long-term psychological stress of HCWs, which may have acute or chronic health effects [13].

In the last decades, a wide body of research has evidenced the disrupting role that major stressful events play on psychological and emotional health, even when individuals are not directly exposed to stressful events [14–16]. Studies conducted during the main outbreaks from SARS [17] to the Middle East respiratory syndrome (MERS) [18], and currently COVID-19, showed that front-line medical staff reports high levels of stress that result in depression, anxiety, and post-traumatic stress disorder (PTSD) due to the outbreaks [19–26]. The overall mental health status of medical personnel responding to new coronavirus pneumonia is generally worse than that of the norm group in China [27]. A psychological survey [28] suggested that the rates of depression, anxiety, insomnia, and stress symptoms among medical staffs involved in epidemic prevention and control were as high as 50.7%, 44.7%, 36.1%, and 73.4% respectively. In addition, studies have shown that the psychological stress scores of medical staff in isolation wards are generally higher [29] and that nurses show more psychological distress in clinical work [30], including physical symptoms such as dizziness, headache, and breathing difficulties [31, 32]. A further Chinese survey reports that HCWs, especially nurses and women who worked in red-zones and front-line, experienced more severe degrees of all measurements of mental health symptoms than others [33]. Finally, a recent meta-analysis reported that the most common indicators of psychological impact reported across studies were anxiety and depression, and the respective prevalence was 33% (28%-38%) and 28% (23%-32%) [25]. Such psychological manifestations can lead to adverse overcrowding of hospital emergency departments [34, 35], causing added workload to the already constrained healthcare systems [36]. Furthermore, distressed staff saw psychological healthcare services as important resources to alleviate acute psychological health disturbances and improve their physical health perceptions [37–40]. Thus, the mental health of HCWs should be carefully considered by hospital managers and healthcare authorities [20, 41, 42]. Despite it is important to understand the prevalence and patterns of the psychological impact of the COVID-19 pandemic on HCWs, to date there are only two studies that have investigated this in Italy. These Italian studies are in line with previous reports from China, confirming a substantial proportion of mental health issues, particularly among young women and front-line HCWs [43, 44].

We conducted an online survey to explore the psychological stress status and psychological care needs of the HCWs in Italy during the COVID-19 outbreak period, between March and May 2020. The aim of the study is twofold: (a) to describe psychological symptoms (i.e. depression, anxiety, somatization, and post-traumatic symptoms), sociodemographic (i.e. age, gender), and workload (i.e. location, working position, experience with patient death) characteristics among the different healthcare professions; and (b) to explore whether and to what extent psychological symptoms, sociodemographic and workload characteristics are associated with psychological care needs. Based upon previous literature we expected that: (a) women, younger and personnel with a heavier workload, would exhibit more symptoms of psychological pressure than all other HCWs; and (b) psychological symptoms, sociodemographic and workload characteristics would be the predictors of psychological care needs.

## 2. Materials and methods

### 2.1 Participants and procedure

A sample of 1,223 HCWs was recruited using an online survey, from March 30th to May 3rd, 2020. The recruitment period coincided with the peak of the COVID‑19 epidemic when strict lockdown measures for all people in the country were in place. An online survey was compiled and completed through the Qualtrics platform (https://www.qualtrics.com). Participants were recruited through social network communities using snowball sampling. The survey took approximately 20 minutes to be completed. To optimize ecological validity, all HCWs from 18 to 65 years old were included, considering that this range represents the minimum and maximum ages to be legally employed as HCWs in Italy. Inclusion criteria were being at least 18 years old and working in the National Health Service. After removing those who did not satisfy the inclusion criteria, the final sample was composed by 933 (76.3%) participants [76.5% women; mean age was 41.77 (sd = 12.08, median = 41.00); 73% postgraduate] from Italian regions (sample distribution: north N = 359, 38.5%, central N = 145, 15.5%, south N = 429, 46%). The sample included 24% physicians, 42.3% nurses, 10.6% technicians from radiology and laboratory medicine, 17.7% Unlicensed Assistive Personnel (UAP), 5.4% other hospital staff (e.g. pharmacists and ambulance drivers). All participants completed the survey anonymously and provided online informed consent to participate. Participants were informed about privacy, ethical aspects and data treatment and they could cease the process at any time. The study was designed and carried out in accordance with the World Medical Association Declaration of Helsinki and its subsequent revisions [45] and approved by the Ethics Committee of the Department of Psychological, Health and Territorial Sciences (DiSPuTer) of University G.d'Annunzio—Chieti-Pescara.

### 2.2 Measures

#### 2.2.1 Sociodemographic characteristics and exposure to COVID-19

Ad-hoc questions concerning sociodemographic and occupational variables were included in the online survey. Data were self-reported by the participants, including gender, age, educational level, workload characteristics (i.e. working position, geographic location, and patients' death), and different healthcare professions (physician, nurse, technicians from radiology and laboratory medicine, UAP, and other hospital staff). Working position, that is whether they were (front-line) or not (second-line) directly involved in the clinical management of patients with suspected or confirmed COVID-19; geographic location, that is whether participants worked in one of the regions (Lombardy, Veneto, Piemonte, and Emilia Romagna) declared "red-zones" because of the highest rate of infections and deaths, accounting for the 70% of infected from COVID-19 in Italy [46]; and experience with patient death (namely, at least one of the participant's own cared patient died from COVID-19).

#### 2.2.2 Perception of psychological care needs

Perception of psychological care needs was assessed with a single item by asking whether participants were explicitly experiencing the need for receiving some forms of psychological help because of the epidemic-related situation. Answers were based on four choices: "Yes, and I asked for help from my therapist/doctor of trust", "Yes, and I have sought psychological help from a specialist", "Yes, but I did not ask for help"; "No, I do not have psychological care needs". According to these answers, participants were divided into 2 groups, those who felt the need for psychological help (first 3 options) (n = 284, 39.3%), and those who did not.

#### 2.2.3 Depression symptoms

Depression symptoms were measured using the Patient Health Questionnaire (PHQ-9), a 9-item self-report measure designed to screen for depression in primary care and other clinical settings [47]. The PHQ-9 items assess the presence of sad, empty, or guilt, accompanied by somatic and cognitive changes (e.g., low energy, sleeping trouble, concentration difficulties) that significantly affect the individual’s capacity to function. Subjects were asked to report the presence of each symptom during the last two weeks on a 4-point rating scale from 0 ("not at all") to 3 ("nearly every day"). The total PHQ-9 scores range from 0 to 27, scores of <5 represent the absence of depression symptoms, and higher scores indicating greater severity of depression. The PHQ-9 is widely used in clinical and research settings and be provided with sound psychometric characteristics [48]. Within this sample, Cronbach's α was 0.88.

#### 2.2.4 Anxiety symptoms

Anxiety symptoms were assessed using the Generalized Anxiety Disorder scale (GAD-7), a 7-item self-report questionnaire that is widely used in clinical and research settings for screening anxiety [49]. Anxiety symptoms include, for example, excessive fear, feeling nervous, trouble relaxing, and anticipation of future threats. Participants were asked to rate how often they have been bothered by each symptom during the past two weeks. Responses are scored on a 4-point rating scale from 0 ("not at all") to 3 ("every day"). Total scores range from 0 to 21, scores of <5 represent the absence of anxiety symptoms, and higher scores reflecting higher severity levels of generalized anxiety disorder symptomology. The GAD-7 has good reliability, construct, factorial, and procedural validity [50, 51]. Within this sample, Cronbach's α was 0.91.

#### 2.2.5 Somatization symptoms

Somatization was measured using the self-report 15-item Patient Health Questionnaire [52]. The PHQ-15 is widely used as a screening instrument for somatization in different healthcare settings. Somatization is one of the most common issues in healthcare services, associated with substantial functional impairment and healthcare utilization. The PHQ-15 items assess the presence of one or more somatic symptoms that are distressing (e.g., fatigue, pain, gastrointestinal symptoms) [52]. Participants were asked to rate the severity of 15 symptoms as 0 ("not bothered at all"), 1 ("bothered a little"), or 2 ("bothered a lot") during the last 4 weeks. Thus, the total PHQ-15 score ranges from 0 to 30, scores of <5 represent the absence of somatization symptoms, and higher scores reflecting higher severity levels of somatization symptoms. Within this sample, Cronbach's α was 0.84.

#### 2.2.6 Post-traumatic stress disorder symptoms

The psychological impact of the COVID-19 pandemic was measured using the Impact of Event Scale-Revised (IES-R) Italian version [53], a widely used measure of psychological distressing symptoms due to a specific stressful event [54]. Post-traumatic stress disorder symptoms include symptoms in which exposure to a traumatic or stressful event is expressed as an internalizing (e.g., intrusive distressing memories of the traumatic event) or externalizing (e.g., irritability or anger) behaviour. The 22-item scale produces three subscale scores for assessing, namely intrusive thoughts (IES-IT), hyperarousal (IES-H), and avoidance symptoms (IES-A) in the previous 7 days concerning the event. Items are rated on a 5-point scale ranging from 0 ("not at all") to 4 ("extremely"). The IES-R yields a total score ranging from 0 to 88, with scores of 33 or higher reflect probable PTSD [55]. Within this sample, Cronbach's α was 0.92 for the total scale, 0.85 for the intrusive thoughts, 0.80 for the hyperarousal, and 0.76 for the avoidance subscales.

### 2.3 Statistical analysis

Chi-square test (χ^2^) and analysis of variance (ANOVA) were used for evaluating sociodemographic and clinical variables in between-group comparison of physicians, nurses, technicians, UAP, and other hospital staff. Student’s t-test and chi-square test (χ^2^) were the standard statistical analyses used to compare clinical variables in between-group differences for gender, age, geographic location, working position, experience with patient’s death and psychological care needs. The standardized mean difference was used as a measure of effect size (Cohen’s d). A Cohen’s d of 0.20–0.50 is considered small, 0.50–0.80 moderate, and >0.80 large [56]. Tukey’s post hoc test, called HSD-honestly significant difference, is an indicator which, in multiple comparison statistics, likens the means of all groups to the mean of every other group and is considered the best available method in cases when confidence intervals are desired or if sample sizes are unequal [57]. Binary logistic regression analysis was performed to identify major determinants that best predict psychological care needs in participants. Psychological care needs were considered as a dependent variable (dummy coded: 0 = absence of psychological care needs; 1 = presence of psychological care needs) and the independent variables were age, gender, patients’ death, location, working position, post-traumatic symptoms, depression, anxiety, and somatization. We aimed to determine the extent to which each sociodemographic (age, gender, experience with patient death, location, and working position) and clinical (post-traumatic symptoms, depression, anxiety, and somatization) factor was able to significantly add to explain the variance of psychological care needs.

All statistical analyses were computed using IBM SPSS Statistics version 25 [58].

## 3. Results

Table 1 reports the sociodemographic and workload characteristics of the healthcare profession subgroups. Gender was not equally represented in the sample and women were significantly more prevalent than men in all professional categories (χ2 = 35.97, p < .001). To a closer look, standardized deviates indicate that more men and fewer women were represented within physicians when compared to other healthcare profession subgroups. The number of workers who worked in the red zone was lower than those working in non-red-zone areas. Compared with other participants, more UAP and technicians were working in the red-zones (χ2 = 31.34, p < .001). Front-line workers were more prevalent in the sample (76%). Other hospital staff members and UAP, as expected, were more present in second-line healthcare units than other professionals (χ2 = 27.37, p < .001). The number of HCWs who experienced at least one of their patient who died from COVID-19 (40.6%) was significantly lower than who did not experience that. As also expected, other hospital staff members experienced even lower patient deaths compared to other categories (i.e., physicians, nurses, technicians, and UAP) (χ2 = 21.48, p < .001).

**Table 1:** Sociodemographic and workload characteristics of the healthcare profession subgroups.

	N (%)							
Variables	Total sample	Physicians	Nurses	Technicians	UAP	Other hospital staff	χ2	p
**Overall**	933	224 (24)	395 (42.3)	99 (10.6)	165 (17.7)	50(5.4)		
**Gender**								
Men	219 (23.4)	85 (37.5)	70 (17.7)	19 (19.2)	31 (18.8)	14 (28)	35.97	< .001
Women	714 (76.5)	139 (62.1)	325 (82.3)	80 (80.8)	134 (81.2)	36 (72)		
**Age**								
≤40	454 (48.7)	99 (44.2)	201 (50.8)	58 (58.6)	67 (40.6)	29 (58.0)	12.50	0.02
>40	479 (51.3)	12 5(55.8)	194 (49.1)	41 (41.4)	98 (59.4)	21 (42.0)		
**Location**								
Red-zone	305 (32.7)	62 (27.7)	110 (27.8)	45 (45.5)	77 (46.7)	11 (22)	31.34	< .001
No-red-zone	628 (67.3)	162 (72.3)	285 (72.2)	54 (54.5)	88 (53.3)	39 (78)		
**Working position**								
Front-line	709 (76)	180 (80.4)	307 (77.7)	84 (84.8)	111 (67.3)	27 (54)	27.37	< .001
Second-line	224 (24)	44 (19.6)	88 (22.3)	15 (15.2)	54 (32.7)	23 (46)		
**Patients’ death**								
Yes	379 (40.6)	92 (41.1)	172 (43.5)	44 (44.4)	66 (40)	5 (10)	21.48	< .001
No	554 (59.4)	132 (58.9)	223 (56.5)	55 (55.6)	99 (60)	45 (90)		

Table 2 shows the between-group descriptive statistics of psychological symptoms, and psychological care needs in total sample and healthcare profession subgroups. No significant difference between the healthcare profession subgroups was found, except for somatization symptoms. Nurses and UAP showed significantly higher somatic symptoms than physicians (F(1,720) = 7.17, p < .05). Of note, 71% (N = 515) of the sample scored above the PHQ-15 cut-off, suggesting that majority of the sample was suffering from somatic symptoms. Moreover, IES-R scores show that 55% (N = 482) of the participants suffered from distress. Specifically, nurses experienced more intrusive thoughts (IES-IT) than other hospital staff members (F_(1,720)_ = 2.92, p < .05).

**Table 2:** Psychological symptoms and psychological care needs in total sample and healthcare profession subgroups.

	Total sample	Physicians	Nurses	Technicians	UAP	Other hospital staff	F/χ^2^	p	Tukey post hoc test
**PHQ-9**									
Mean (SD)	6.71 (5.66)	6.44 (5.58)	7.01 (6.02)	7.06 (5.25)	6.53 (5.38)	5.36 (4.53)	1.04	.38	-
Positives^a^	453 (57.9%)	105 (54.4%)	200 (59.3%)	52 (62.7%)	77 (59.7%)	19 (46.3%)	4.44	.35	
**GAD-7**									
Mean (SD)	7.55 (5.65)	7.23 (5.39)	8.05 (5.95)	7.48 (5.54)	7.24 (5.42)	6.11 (5.11)	1.64	.16	-
Positives	525 (65.2%)	124 (63.6%)	232 (67.2%)	57 (66.3%)	88 (65.2%)	24 (54.5%)	3.10	.54	
**PHQ-15**									
Mean (SD)	8.28 (5.46)	6.67 (4.92)	8.89 (5.57)	8.56 (5.15)	9.47 (5.89)	6.92 (4.51)	7.17	< .001	Nurse > Physician; UAP > Physician
Positives	515 (71%)	111 (60%)	243 (76.9%)	54 (72%)	81 (71.7%)	26 (72.2%)	16.31	.003	
**IES-R**									
Mean (SD)	4.92 (2.18)	4.69 (2.18)	5.15 (2.15)	4.93 (2.04)	4.90 (2.26)	4.19 (2.25)	2.86	.02	Nurse > Other hospital staff
Positives	482 (55%)	109 (50.5%)	223 (61.1%)	50 (53.8%)	83 (52.9%)	17 (37%)	13.67	.008	
** IES-IT**									
Mean (SD)	1.85 (0.88)	1.78 (0.85)	1.94 (0.87)	1.86 (0.87)	1.82 (0.90)	1.52 (0.88)	2.92	.02	Nurse > Other hospital staff
**IES-H**									
Mean (SD)	1.65 (0.85)	1.57 (0.86)	1.73 (0.85)	1.68 (0.83)	1.64 (0.88)	1.42 (0.82)	2.16	.07	
**IES-A**									
Mean (SD)	1.42 (0.69)	1.34 (0.73)	1.48 (0.66)	1.39 (0.64)	1.44 (0.72)	1.25 (0.71)	2.06	.08	-
**Psychological care needs**									
Positives	284 (39.3%)	67 (36.2%)	133 (42.2%)	25 (33.8%)	42 (37.2%)	17 (47.2%)	3.97	.41	

^a^ Positives: scoring higher than the following cutoff scores = PHQ-9 < 5, GAD-7 < 5, PHQ-15 < 5, IES-R < 33.

Table 3 reports differences in psychological symptoms and psychological care needs between gender and age ranges. Women scored higher in all the psychological variables, effect sizes in the moderate-to-large range indicate that female care workers were experiencing higher levels of anxiety (d = 0.50) and somatization symptoms (d = 0.82) than men. Also, more women stated they needed psychological care more than men (χ2 = 28.06, p < .001). Finally, younger participants (aged <40 years-old) were experiencing higher levels of somatization, depression, anxiety, and PTSD symptoms (effects size in the small range, from d = 0.22 to d = 0.31).

**Table 3:** Psychological symptoms and psychological care needs in total sample and subgroups.

		Gender					Age				
	Total sample	Men (n = 219)	Women (N = 714)	t/χ2	p	d	≤40 (N = 454)	>40 (N = 479)	t/χ2	p	d
**PHQ-9**											
Mean (SD)	6.71 (5.66)	4.96 (5.20)	7.24 (5.68)	4.88	< .001	0.41	7.58 (5.82)	5.99 (5.43)	3.94	< .001	0.29
Positives^a^	453 (57.9%)	80 (42.2%)	373 (62.7%)	25.11	< .001		229 (64.7%)	224 (52.2%)	12.37	< .001	
**GAD-7**											
Mean (SD)	7.55 (5.65)	5.44 (5.05)	8.19 (5.66)	6.02	< .001	0.50	8.36 (5.54)	6.87 (5.66)	3.75	< .001	0.27
Positives	525 (65.2%)	88 (45.3%)	437 (71.4%)	44.39	< .001		268 (73.2%)	257 (58.5%)	18.96	< .001	
**PHQ-15**											
Mean (SD)	8.28 (5.46)	5.08 (4.13)	9.28 (5.43)	9.41	< .001	0.82	9.19 (5.71)	7.53 (5.14)	4.11	< .001	0.31
Positives	515 (71%)	86 (48.6%)	429 (78.1%)	56.80	< .001		254 (77.2%)	261 (65.9%)	11.14	.001	
**IES-R**											
Mean (SD)	4.92 (2.18)	4.15 (2.13)	5.15 (2.15)	5.86	< .001	0.47	5.25 (2.15)	4.63 (2.18)	4.19	< .001	0.29
Positives	482 (55%)	83 (39.2%)	399 (59.8%)	28.06	< .001		246 (61%)	236 (49.8%)	11.14	.001	
** IES-IT**											
Mean (SD)	1.85 (0.88)	1.53 (0.83)	1.94 (0.87)	6.12	< .001	0.48	1.95 (0.89)	1.76 (0.85)	3.25	.001	0.22
**IES-H**											
Mean (SD)	1.65 (0.85)	1.35 (0.79)	1.75 (0.85)	5.99	< .001	0.48	1.77 (0.84)	1.55 (0.85)	3.84	< .001	0.26
**IES-A**											
Mean (SD)	1.42 (0.69)	1.28 (0.72)	1.46 (0.68)	3.33	.001	0.26	1.52 (0.68)	1.32 (0.69)	4.36	< .001	0.29
**Psychological care needs**											
Positives	284 (39.3%)	37 (20.6%)	247 (45.2%)	35.14	< .001		156 (54.9%)	128 (45.1%)	17.25	< .001	

^a^ Positives: scoring higher than the following cutoff scores = PHQ-9 < 5, GAD-7 < 5, PHQ-15 < 5, IES-R < 33.

Working in a high infected area (red-zones) and directly with COVID-19 patients (front-line) affected the psychological health of participants to a smaller degree (Table 4). Effect sizes in the small range have been found for depression (d = 0.19) and intrusive thoughts (d = 0.14) in front-line workers, and distressing symptoms in HCWs working in the red-zones (Table 4). Conversely, a major impact was found in participants who experienced the death of one of their patients. HCWs who lost one of their patients reported higher levels of depression (d = 0.22), anxiety (d = 0.19), and PTSD symptoms (d = 0.30), including intrusive thoughts (d = 0.32), hyperarousal (d = 0.25), and avoidance symptoms (d = 0.22). As a likely consequence, they reported a higher need for psychological care than those who did not have the same experience (χ2 = 6.23, p < .01).

**Table 4:** Psychological symptoms and psychological care needs in total sample and subgroups.

		Location					Working position					Patients’ death				
	Total sample	Red-zone	No-red-zone	t/χ2	p	d	Front-line	Second-line	t/χ2	p	d	Yes	No	t/χ2	p	d
**PHQ-9**																
Mean (SD)	6.71 (5.66)	7.42 (5.72)	6.37 (5.61)	2.43	.01	0.19	6.90 (5.71)	6.08 (5.47)	1.72	.08	0.15	7.33 (5.69)	6.10 (5.58)	3.06	.002	0.22
Positives^a^	453 (57.9%)	155 (61.5%)	298 (56.1%)	2.03	.15		355 (58.9%)	98 (54.4%)	1.11	.29		209 (64.9%)	244 (52.9%)	11.15	.001	
**GAD-7**																
Mean (SD)	7.55 (5.65)	8.07 (5.50)	7.30 (5.71)	1.81	.07	0.14	7.72 (5.65)	6.97 (5.65)	1.58	.11	0.13	8.08 (5.57)	7.02 (5.69)	2.68	.008	0.19
Positives	525 (65.2%)	188 (72.9%)	337 (61.6%)	9.79	.002		413 (66.8%)	112 (59.9%)	3.04	.08		238 (72.3%)	287 (60.3%)	12.44	< .001	
**PHQ-15**																
Mean (SD)	8.28 (5.46)	8.50 (5.21)	8.18 (5.58)	0.73	.46	0.06	8.19 (5.48)	8.62 (5.42)	0.90	.36	0.08	8.60 (5.51)	7.97 (5.41)	1.55	.12	0.12
Positives	515 (71%)	173 (74.9%)	342 (69.2%)	2.45	.11		395 (70.7%)	120 (72.3%)	0.16	.68		229 (76.1%)	286 (67.5%)	6.36	.01	
**IES-R**																
Mean (SD)	4.92 (2.18)	5.10 (2.18)	4.83 (2.18)	1.75	.08	0.12	5.06 (2.21)	4.47 (2.04)	3.40	.001	0.27	5.25 (2.16)	4,60 (2.16)	4.45	< .001	0.30
Positives	482 (55%)	166 (58.7%)	316 (53.2%)	2.30	.12		385 (57.6%)	97 (46.4%)	8.10	.004		236 (65.9%)	246 (47.4%)	29.36	< .001	
**IES-IT**																
Mean (SD)	1.85 (0.88)	1.93 (0.91)	1.81 (0.86)	1.92	.05	0.14	1.90 (0.89)	1.69 (0.81)	3.02	.003	0.24	1.99 (0.87)	1.71 (0.86)	4.84	< .001	0.32
**IES-H**																
Mean (SD)	1.65 (0.85)	1.71 (0.85)	1.63 (0.86)	1.42	.15	0.09	1.70 (0.86)	1.51 (0.81)	2.78	.006	0.22	1.76 (0.84)	1.55 (0.85)	3.78	< .001	0.25
**IES-A**																
Mean (SD)	1.42 (0.69)	1.46 (0.67)	1.39 (0.70)	1.33	.18	0.10	1.46 (0.69)	1.27 (0.67)	3.48	.001	0.28	1.49 (0.69)	1.34 (0.68)	3.23	.001	0.22
**Psychological care needs**																
Positives	284 (39.3%)	98 (42.4%)	186 (37.8%)	1.40	.23		224 (40.2%)	60 (36.1%)	0.88	.34		134 (44.7%)	150 (35.5%)	6.23	.01	

^a^ Positives: scoring higher than the following cutoff scores = PHQ-9 < 5, GAD-7 < 5, PHQ-15 < 5, IES-R < 33.

As shown in Table 5, HCWs who needed psychological care scored dramatically higher to all psychological variables, as suggested by size effects in the large range for depression (d = 0.96), anxiety (d = 1.00), somatic symptoms (d = 0.84), and distress (d = 0.96). Moreover, from 76.4% to 87.7% HCWs who perceived the need for psychological support reported a clinical alarming level in all the psychological scales.

**Table 5:** Psychological symptoms in total sample and psychological care needs subgroups.

		Psychological care needs				
	Total sample	Yes	No	t/χ2	p	d
**PHQ-9**						
Mean (SD)	6.71 (5.66)	9.71 (6.15)	4.76 (4.43)	11.73	< .001	0.96
Positives^a^	453 (57.9%)	232 (81.7%)	185 (42.1%)	110.49	< .001	
**GAD-7**						
Mean (SD)	7.55 (5.65)	10.54 (5.42)	5.51 (4.81)	12.73	< .001	1.00
Positives	525 (65.2%)	249 (87.7%)	216 (49.2%)	111.21	< .001	
**PHQ-15**						
Mean (SD)	8.28 (5.46)	10.85 (5.54)	6.61 (4.73)	10.62	< .001	0.84
Positives	515 (71%)	245 (86.3%)	268 (61%)	53.21	< .001	
**IES-R**						
Mean (SD)	4.92 (2.18)	44.13 (14.84)	30.21 (14.30)	12.59	< .001	0.96
Positives	482 (55%)	217 (76.4%)	173 (39.4%)	95.02	< .001	
**IES-IT**						
Mean (SD)	1.85 (0.88)	2.27 (0.82)	1.55 (0.82)	11.71	< .001	0.88
**IES-H**						
Mean (SD)	1.65 (0.85)	2.10 (0.83)	1.34 (0.75)	12.74	< .001	0.97
**IES-A**						
Mean (SD)	1.42 (0.69)	1.67 (0.68)	1.23 (0.63)	8.92	< .001	0.68

^a^ Positives: scoring higher than the following cutoff scores = PHQ-9 < 5, GAD-7 < 5, PHQ-15 < 5, IES-R < 33.

A binary logistic regression analysis has been performed to assess which variables contributes to explain the need of psychological care in our sample. As reported in Table 6, the model (χ2 = 192.40, gdl = 9, p < .001) showed that women (β = -.69, p < .01; OR = 2.00, 95% CI = 1.25–3.20), psychological distress (β = .17, p < .01; OR = 1.29, 95% CI = 1.04–1.33), anxiety (β = .06 p < .05; OR = 1.06, 95% CI = 1.00–1.12), and depression (β = .06 p < .05; OR = 1.06, 95% CI = 1.00–1.11) accounted for 32% of the variance. Conversely, age, workload characteristics, as well as the experience of patients' death did not significantly affect the need of psychological support.

**Table 6:** Predictors of psychological care needs.

Factors	β	p	OR [95% CI]	R^2^
				.32
**Age**	-.01	.08	0.98 [0.97, 1]	
**Gender**	-.69	.004	2.00 [1.25, 3.20]	
**Patients death**	.08	.71	1.08 [0.72, 1.63]	
**Location**	-.04	.83	0.96 [0.65, 1.41]	
**Working position**	-.03	.91	.97 [0.61, 1.56]	
**IES-R**	.17	.01	1.29 [1, 1.33]	
**GAD-7**	.06	.03	1.06 [1, 1.12]	
**PHQ-9**	.06	.03	1.06 [1, 1.11]	
**PHQ-15**	.03	.12	1.03 [0.99, 1.08]	

## 4. Discussion

COVID-19 pandemic and consequent lockdown have rapidly changed everyone's life and habits, affecting the psychological health of people worldwide. Nonetheless, bound by an oath to heal, HCWs have continued to work, confronting a deadly and highly contagious illness, while the rest of the people were bunkered in homes. At the time of this investigation, Italy was the second most damaged country after China to deal with the new COVID-19, forcing the healthcare industry, services, and professionals to reconsider priorities and face a new challenging dramatic situation that nowadays has unfortunately spread worldwide. While probably years are needed before the mental health toll of the COVID-19 pandemic will be completely understood, the latest research on the wellbeing of healthcare workers is drawing an alarming picture. Besides the fear of infection and the separation from families, HCWs have been reported to suffer from depression, anxiety, insomnia, and distress, as shown by several studies after the COVID-19 first outbreak in China [19–24, 26]. The present study aimed to explore the sociodemographic and psychological pressures of Italian HCWs, assessing psychopathological symptomatology and their needs for specific psychological cares.

We investigated 933 HCWs from different regions in Italy and most of the participants were working in close contact with COVID-19 patients (front-line HCWs) and approximately 1/3 of them in a highly infected area (the so-called red-zones). Moreover, half of the physicians, nurses, technicians, and UPAs declared to have experienced the loss of one of their patients due to the pandemic. Some relevant aspects can be highlighted from the results of our survey.

Overall, from half to two-thirds of Italian HCW participants experienced a high level of psychological distress as shown by the proportion of HCWs scoring above the cutoff for clinical attention to scales assessing depressive, anxiety, post-traumatic stress disorder, and somatization symptoms. Even though we expected high levels of distress in our sample, surprisingly the wide proportion of workers scored within the alarm psychopathological range of scales. This should be considered a relevant warning for future psychological consequences in their post-acute epidemic psychosocial adjustment.

It is also worth noting that the psychological burden of caregiving was not different according to the professional role in the Health Care System. Regardless of being physicians, nurses, technicians, UAP, or other hospital staff members, all healthcare providers experienced a high level of psychological distress during the COVID-19 epidemic, suggesting that high personal and emotional involvement in facing this challenging period was felt by all HCWs and can be at cost for their psychological health in the next future. Although not confirmed by meta-analyzing data [25], our finding is consistent with other studies showing a different psychological impact of COVID-19 among healthcare workers, the general public, and patients.

It is also interesting to note that the experience of somatization symptoms was less present within physicians, even if their psychological stress was similar to people in the other health care professions subgroups. It may be speculated that physicians have some specific resilience to somatization that are likely related to personal accomplishment [59], professional experience, and self-awareness [60]. The recent Luo et al. [25] meta-analysis interestingly found that having sufficient medical resources, as well as up-to-date and accurate health information, constitute a protective factor preventing higher experience of psychological distress. Though speculative since requires more deep qualitative data, an alternative explanation may be that physicians are more familiar with using defense mechanisms as intellectualization and denial as an effective coping response to professional-related distress.

Another relevant finding from our survey is that women were over-represented within our sample and those who were suffering the most because of the burden due to the present emergency. As expected, and in line with previous studies, women confronting with the dramatic working situation were more subject to experience symptoms of depression, anxiety, somatization, and post-traumatic distress than men. Research studies have indeed consistently reported higher prevalence and severity of depression, anxiety [61], somatic symptoms [62], and overall distress [63] in women. In particular, the moderate-to-large effects sizes of the association between IES-R subscales and gender suggest that female HCWs may be at greater risk to develop post-traumatic stress disorders than men. Furthermore, contemporary studies on the health outcomes in HCWs dealing with the COVID-19 show a substantial proportion of mental health issues, particularly among women [25, 33, 43, 44]. On one hand, because of the Italian cultural traditional-bound double roles of women in the family, child-caring, and professional jobs, women may have suffered more than their male colleagues the pressure of working in the COVID-19 emergency. On the other hand, generally, women give more importance to their own internal experiences and others' emotional states than men. Moreover, the growing body of gender-specific studies highlights the relative underuse of health-service and symptom reporting by men [64, 65]. This may suggest a different gender-specific model of interaction with patients [66, 67]. Hence, the gender-related stress shown by female HCWs should be considered as a strong warning message for the psychosocial cost of the re-adjustment to their usual life and a socioeconomic cost for the national healthcare system. Moreover, these outcomes call for the implementation of research in gender-specific medicine to implement psychological programs and to prevent and address different gender and work-related needs and risk factors.

On the same line, younger workers reported higher levels of psychological pressure symptoms compared to their older colleagues. Earlier studies reported that less long-standing experienced HCWs have been found as more vulnerable to emotional distressing symptoms than their older colleagues [68, 69]. Therefore, it can be speculated that Italian younger HCWs are less experienced with difficult, complex, and life-challenging clinical situations than their older and more experienced colleagues, regardless of their professional role in the healthcare system. Contemporary studies on the health outcomes in HCWs confronting the COVID-19 show higher levels of insomnia and negative sleep partners in younger physicians [70] and higher levels of stress in younger health care workers [71]. These results suggest that gender and age could be considered as risk factors for health consequences in hospital and health care facilities workers.

In contrast to our hypothesis, working in front-line directly with COVID-19 infected patients and in the highly infected regional red-zones did not influence more psychopathological symptomatology than working not directly with COVID-19. Conversely, HCWs who experienced the death of one of their COVID-19 infected patients showed higher levels of psychological suffering. Interestingly, the "objective" stress level due to the professional role (i.e., working in highly stressful work environments and more dangerous geographical areas) had less impact on emotional symptoms than the "subjective" stress level due to the loss experience. This finding is however consistent with previous studies. Even if the loss of a patient is an event that should be taken into account in any medical setting, particularly in emergency departments, it has been shown as one of the most common sources of stress for physicians, surgeons, and nurses [72, 73]. Although not completely unexpected, these findings highlight once again the need for psychological support for HCWs. The patient's death may indeed remain an unexpressed feeling, particularly if over-burdened by guilt and a sense of professional failure, which can affect the efficacy of physicians and other HCWs work with patients, with severe health, social and economic costs.

To our knowledge, this is the first report that explored the need for psychological care in the context of the COVID-19 epidemic in Italy. More than one third (39.3%) of HCWs in our sample reported their explicit need for psychological support and 76% to 88% of them showed scores warning for clinical attention to all psychological scales. Strictly related to our findings discussed above, the need for psychological help was explained at 32% mostly by reporting psychological symptoms of distress which were associated with the experience of own patients' death. Once again, if further confirmed, our findings strongly suggest understanding risk factors, such as gender and age, predisposing to poor psychological health and to provide early psychological support to health care workers.

## 5. Limitations and conclusion

Some limitations are to be acknowledged. First, our sample of HCWs included an unbalanced proportion between females and males. Women were over-represented in all professional categories, but between physicians, males were more prevalent. Although our results can not be easily generalized to the overall population, this is a reliable picture of the gender-biased prevalence in healthcare professions in Italy. Second, the cross-sectional nature of our data does not allow us to establish the direction of causality, and longitudinal studies are needed to evaluate whether the current stressful condition will be persistent and the consequences it may have on the health status of HCWs. Third, all the questionnaires we used have been validated and shown excellent psychometric properties in the usual administration. However, research has shown that self-completed questionnaires, electronic, and online administration can be used interchangeably for research [74, 75]. Finally, the online administration of a survey is subject to responder bias. Particularly with HCWs during the dramatic pandemic, people who agreed to answer questions on their psychological health might be much more motivated to participate if psychologically distressed. This can surely limit the generalization of our findings by overestimating psychological distress and the need for psychological care.

Overall, this study shows that HCWs during the COVID-19 outbreak in Italy experienced a high burden of psychological distress, may be at risk for future psychological health-related consequences, and declared their need for psychological support, particularly if women and younger. Our findings highlight that national health authorities are informed about the psychological impact of the epidemic on the psychological health of Italian HCWs and strongly suggest establishing psychological support services for providing adequate professional care for them. Italian media have often called HCWs "heroes" during the most difficult periods of the national epidemic-related quarantine. This label comes at a cost and HCWs are showing their human nature of fragile heroes.

The present study also rises another relevant problem. Physicians, nurses, technicians, and UAP's distress can be a risk, even beyond the close individual level and it may impact directly their patients' health. Distressed HCWs showed less involvement in their relationship with patients [76], to make more medical mistakes [77], and even to compromise the clinical outcomes of patients [78, 79]. The impact of the socio-economic cost of the COVID-19 epidemic might be, therefore, higher and on a larger scale than a single individual psychological health level, thus reinforcing the need for psychological support for HCWs in their daily work.

## Supporting information

S1 Material (SAV)
